# P028: Examining the relationship between fluoroquinolone use and Clostridium difficile infections (CDI): a meta-analysis

**DOI:** 10.1186/2047-2994-2-S1-P28

**Published:** 2013-06-20

**Authors:** MB Formanek, L Herwaldt, ML Schweizer

**Affiliations:** 1 Epidemiology/CADRE, University of Iowa College of Public Health/Iowa City Veterans Affairs Medical Center, Iowa City, IA, USA; 2 Internal Medicine/Clinical Quality, Safety, and Process Improvement, University of Iowa College of Medicine/University of Iowa Hospitals and Clinics, Iowa City, IA, USA; 3 Internal Medicine/CADRE, University of Iowa College of Medicine/Iowa City Veterans Affairs Medical Center, Iowa City, IA, USA

## Introduction

The incidence of CDI has increased substantially during the past decade. *C. difficile* is now one of the most important causes of healthcare-associated infections. Antimicrobial use is the primary risk factor for CDI. Several small studies have assessed whether fluoroquinolones increase the risk of CDI more than other antimicrobials.

## Objectives

To systematically review and evaluate all published studies on the relationship between fluoroquinolone use and CDI.

## Methods

We performed systematic literature searches in PubMed, Wiley’s Cochrane Database of Systematic Reviews (CDSR), Wiley’s Database of Abstracts of Reviews of Effects (DARE), Wiley’s Cochrane Central Register of Controlled Trials (CENTRAL), Scopus (including EMBASE abstracts), and http://ClinicalTrials.gov. We ran the searches on October 15, 2012 with no limits on date or language. We used a random-effects model to obtain meta-analysis summary estimates. After reviewing 431 article abstracts and reviewing 22 articles in detail, we pooled risk estimates from 17 independent study populations.

## Results

When we pooled the crude data from all 17 studies in a random-effects model, fluoroquinolone use was significantly associated with a higher risk of CDI than other antimicrobials (pooled OR: 2.93; 95% CI: 2.12, 4.05). Twelve of 17 studies provided adjusted risk estimates. When we pooled the adjusted data, fluoroquinolone use remained a significant risk factor for CDI, albeit with a lower pooled OR (pooled OR: 1.71; 95% CI: 1.48, 1.96).

## Conclusion

Fluoroquinolone use was associated with increased risk of CDI compared with other antimicrobials. Antibiotic stewardship campaigns to limit to overuse of fluoroquinolones may decrease the incidence of healthcare-associated CDI.

## Disclosure of interest

None declared

